# Food-Origin Mycotoxin-Induced Neurotoxicity: Intend to Break the Rules of Neuroglia Cells

**DOI:** 10.1155/2021/9967334

**Published:** 2021-09-28

**Authors:** Xingyao Pei, Wenjuan Zhang, Haiyang Jiang, Dingkuo Liu, Xinyu Liu, Liuan Li, Cun Li, Xilong Xiao, Shusheng Tang, Daowen Li

**Affiliations:** ^1^Department of Pharmacology and Toxicology, College of Veterinary Medicine, China Agricultural University, Yuanmingyuan West Road No. 2, Haidian District, Beijing 100193, China; ^2^Tianjin Key Laboratory of Agricultural Animal Breeding and Healthy Husbandry, College of Animal Science and Veterinary Medicine, Tianjin Agricultural University, Jinjing Road No. 22, Xiqing District, Tianjin 300384, China; ^3^Tianjin Key Laboratory of Biological Feed Additive Enterprise, S&E Burgeoning Biotechnology (Tianjin) Co., Ltd, Tianjin 300383, China; ^4^State Key Laboratory of Medicinal Chemical Biology and Tianjin Key Laboratory of Molecular Drug Research, College of Pharmacy, Nankai University, Haihe Education Park, Tongyan Road No. 38, Tianjin 300353, China

## Abstract

Mycotoxins are key risk factors in human food and animal feed. Most of food-origin mycotoxins could easily enter the organism and evoke systemic toxic effects, such as aflatoxin B1 (AFB1), ochratoxin A (OTA), T-2 toxin, deoxynivalenol (DON), zearalenone (ZEN), fumonisin B1 (FB1), and 3-nitropropionic acid (3-NPA). For the last decade, the researches have provided much evidences in vivo and in vitro that the brain is an important target organ on mycotoxin-mediated neurotoxic phenomenon and neurodegenerative diseases. As is known to all, glial cells are the best regulator and defender of neurons, and a few evaluations about the effects of mycotoxins on glial cells such as astrocytes or microglia have been conducted. The fact that mycotoxin contamination may be a key factor in neurotoxicity and glial dysfunction is exactly the reason why we reviewed the activation, oxidative stress, and mitochondrial function changes of glial cells under mycotoxin infection and summarized the mycotoxin-mediated glial cell proliferation disorders, death pathways, and inflammatory responses. The purpose of this paper is to analyze various pathways in which common food-derived mycotoxins can induce glial toxicity and provide a novel perspective for future research on the neurodegenerative diseases.

## 1. Introduction

Mycotoxins spontaneously produce in food and feed, which have threatened human and animal health around the world. The thorny issue is not just their toxicity but their high prevalence in agriculture and strong connection with human diet and animal life [1]. Mycotoxins are the most likely toxins to invade human body due to the changes in the natural environment or improper manual handling which may facilitate the occurrence and spread of mycotoxins among food crops and their derivative products [2]. The mycotoxins enter the human body primarily along with plant-based foods in a direct route, and they also come from various food of mycotoxin infected animal origin in an indirect aspect with secondary contamination [3–6]. Thus, mycotoxins could enter every stage of the food chain and every step of food handling through contaminated source to infect final consumers and users. According to official statistics, more than 25% of the global crops are growing fungi and contaminated with mycotoxins [7]. In addition, an increasingly volatile global climate as a force majeure factor may also aggravate the intensity of mycotoxins growth [8]. Early studies have shown that large quantities of cereal-based products were contaminated with fusarium toxin particularly in a long period of rainy years [9]. In addition, sugarcane has also been proved to be a carrier of mycotoxins in humid environments. For instance, sugarcane was harvested in the south of China and transported to northern regions for sale: during transport and storage, damp conditions favor growth of Arthrinium spp., which secrete the mycotoxin 3-NPA. The above process has resulted in the established CNS disease known as Mildewed Sugarcane-induced Dystonia, a disease that has been prevalent in eastern regions of China (including Beijing) among children who have eaten sugarcane purchased from street vendors [10, 11]. More remarkably, the extent to which crops are contaminated with mycotoxins has been more unpredictable as the global warming. Also, note that human behavior of contemporary lifestyle like plant hybridization is reducing the stress tolerance of crops and making food and feed more susceptible to mycotoxin exposure, not to mention the high stability of mycotoxins [12].

Mycotoxins are all secondary metabolites of different fungi [13]. Although the mycotoxins have a large population of more than 300 species, the most common mycotoxins in human food and animal feed are aflatoxin B1 (AFB1), ochratoxin A (OTA), T-2 toxin, deoxynivalenol (DON), zearalenone (ZEN), fumonisin B1 (FB1), and 3-nitropropionic acid (3-NPA), which are the protagonist in this paper [14]. Available literatures have listed out acute or chronic toxicity including hepatotoxicity, nephrotoxicity, dermal toxicity, reproductive toxicity, and immunotoxicity in humans and food-producing animals caused by an excessive number of food-origin mycotoxins [15, 16]. As research continues, mycotoxins have been regarded as the typical food-related exogenous toxin which could attack the brain, and some of them have been known to cross the blood-brain barrier (BBB), which could accumulate in the brain and disrupt the normal function of central nervous system (CNS) [17–20]. So far, the neurotoxicity of mycotoxins involved in the predisposing factors of nerve diseases is becoming better recognized by researchers. In general, the AFB1, DON, T-2, OTA, FB1, and 3-NPA, which have strong affinity with brain, could enter the systemic circulation through consumption or contact, and some of their metabolites could easily cross the BBB and reach in the nerve cells via transporters [21–23]. It was important to note that after neurotoxin 3-NPA was absorbed by the stomach and intestines, the BBB was rapidly and extensively destroyed [24]. Furthermore, the fact for mycotoxins to enter the brain and develop toxic effects has been highlighted in recent literatures. For instance, 3-NPA is a mitochondrial neurotoxin that shuts down energy transformation in nerve system, resulting in brain damage that appears clinically in the form of persistent (lifelong) dystonia and ballism [25]. Furthermore, experimental animal models of 3-NPA-induced neurotoxicity have been used to help find therapeutic strategy for Huntington's disease [26, 27]. OTA is a neurotoxin that has a high affinity with the brain; the mice treated with OTA showed acute depletion of both dopamine and metabolites in the striatum with excessive OTA in the brain [26, 27]. OTA also caused male-specific autism in vivo and in vitro by combining the microRNA and X-chromosome in the brain [28]. Recently published studies have revealed that the metallothionein (MTs) gene in the fetal brain could be upregulated by T-2 toxin accumulated in the brain of female mice, demonstrating that the regulation of mycotoxins on antioxidant-related genes could be two-way transmitted between mother brain and fetus brain [22]. However, researchers also found that AFB1 affected neurons potentially by activating immune cells in the bloodstream of mice, causing neurodevelopmental and function abnormalities, in addition to causing brain damage directly [29–31]. Besides, the ability of AFB1 to indirectly affect neurons by affecting the function of glial cells has also been demonstrated [32]. Most importantly, mycotoxins have been demonstrated to increase the occurrence of global neurological diseases. It has been reported that the concentration of T-2 toxin in the serum of 182 residents with a history of mycotoxin exposure was significantly increased, neurodevelopment was hindered, and exercise balance, visual function, reaction speed, cognitive ability, and emotional management ability were all weakened. In addition, the decrease in frontal cortex activity was detected by quantitative EEG [33]. Through individual neuropsychological data and symptom observation, mycotoxins were also suspected to be one of the triggers of autism spectrum disorders, consistent with recent epidemiological research reports on mycotoxins and autism spectrum disorders [34]. A neurophysiological test for children showed that the evoked potentials of brainstem, visual, and somatosensory were abnormal after long-term exposure to mycotoxins [35]. The cognitive impairment of 277 children in Poland was proved by a 6-year mycotoxin exposure test, indicating that the exposure time was negatively related to the IQ of participants. Similarly, the concentration of ochratoxin in urine and serum of 52 autistic patients was significantly higher than that of the control groups. Additionally, the upregulation of microRNA-132 mediated by OTA in vitro has been verified, which was connected with depression-related genes PTEN and MeCP2. Conversely, the way of adsorbing or removing OTA could rescue autism symptoms [36]. Based on the characteristics of these common mycotoxins about their easy passage through the BBB and their potential threat to the central nervous system, exploring the interconnections between their neurotoxic mechanisms and the nerve-associated diseases is becoming more urgent for us.

**Figure 1:**
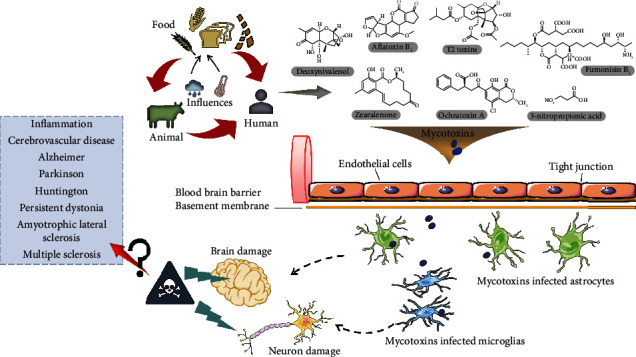
Food-origin mycotoxins cause neuronal and brain damage by infecting astrocytes and microglia. Mycotoxins including aflatoxin B1 (AFB1), ochratoxin A (OTA), T-2 toxins, deoxynivalenol (DON), zearalenone (ZEN), fumonisin B1 (FB1), and 3-nitropropionic acid (3-NPA) enter the body mainly through food crops and animal-origin foods (meat, eggs, milk, sugarcane, and edible viscera). Mycotoxins and its metabolites can easily cross the blood-brain barrier (BBB) and infect astrocytes and microglia, which eventually leading to neuronal damage and brain damage. Whether mycotoxins cause neurodegenerative diseases, such as Alzheimer, Parkinson, Huntington, and amyotrophic lateral sclerosis need further research.

A large number of records indicated that glial cells especially astrocytes and microglia play irreplaceable central roles in brain homeostasis, neural information transmission, neuronal survival, and neuroinflammation [37–40]. Furthermore, astrocytes and microglia are not only the most abundant glial cells to protect CNS but also the crucial participants in both brain injury and neurodegenerative disorders, particularly in the Alzheimer's, Parkinson's, and Huntington's diseases [41]. Although the amount of available information about the mycotoxin-induced gliocytotoxicity are still limited and lack complete integration and analysis, the neurotoxicity through altering the survival status and function of the glia cells have been demonstrated in mycotoxin [42]. Previous research proved that the oxidative stress and gene expression of ZEN-treated neurons were strictly regulated by glial cells with the evidence that the presence or absence of glial cells affected the expression of thyroid receptors *α* and *β* [43]. Another report about typical neurotoxin FB1 suggested that the primary and mature neurons of the mice were not the primary targets of FB1 (50 *μ*mol/L) neurotoxicity in vitro; on the contrary, the immune active cells such as astrocytes showed both obvious cytotoxicity and cell death after 8-day exposure. In addition, the obvious microglial cytotoxicity mediated by FB1 (20 *μ*mol/L) was also observed in mice at the 4th day. Therefore, increasing evidences have indicated that the neurotoxicity of mycotoxins was secondary to the complex roles of glial cells, potentially promoting the development of neurodegenerative diseases [44]. Considering the indispensable roles of glial cells in CNS diseases, as well as the inevitable mycotoxins in feed and food, we now comprehensively describe the essential connection between mycotoxins and glial cells from the perspective of astrocytes and microglia cells (Figure 1). Despite the evidence that ZEN causes glial toxicity is limited, the interferences of other mycotoxins on glial cells were summarized in this report, so as to systematically explain how the mycotoxins stimulate the glial cell status related to the nerve injury. The long-term purpose is to provide necessary information for protecting CNS against the threat of exogenous factors and find an effective way to delay neurodegenerative diseases.

## 2. The Dual Role of Glial Cell Activation

During external stimulus, astrocytes and microglia would transform resting state into activation state, undergo changes in morphology, proliferation, and migration [45]. It has been known that glial cells could play the dual roles of protection and damage depending on different activation levels, which can not only protect and repair the brain but also cause damage to the CNS [46, 47]. The activation and proliferation of glial cells have been proven to affect the microenvironment of nerve tissue repair after injury or ischemia: excessive activation of astrocytes and microglial cells, on the one hand, forms a glial scar and oppresses the microvessels, on the other hand, tends to secrete harmful cytokines, which affect nerve regeneration. Meanwhile, activated glial cells produce a large amount of nitric oxide, which affects DNA modification and lead to neuron injury [48, 49]. Rapid and severe overactivation of glial cells caused by neurotoxic substances could lead to a metabolic disturbance in the interactions between neurons and astrocytes and result in severe brain disease [50]. Related literatures have confirmed that this process has been implicated in the course of Alzheimer, Huntington's dance, demyelinating disease, and brain trauma [51].

### 2.1. Mycotoxin-Mediated Astrocyte Activation

**Figure 2:**
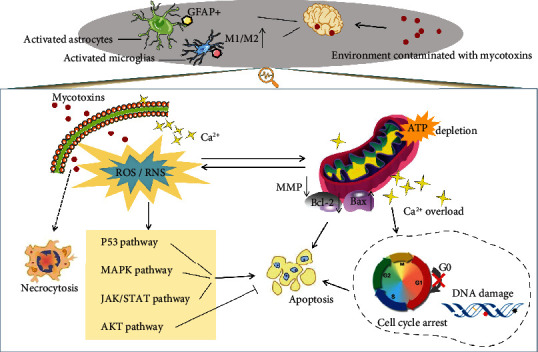
Main pathways of mycotoxin-induced neurotoxicity in astrocytes and microglia. Mycotoxins could cross the blood–brain barrier (BBB) and activated astrocytes and microglia. Mycotoxins stimulate the generation of reactive oxygen species (ROS) and reactive nitrogen species (RNS), lead to mitochondrial dysfunction (decrease of mitochondrial membrane potential (MMP), increase the ratio of Bax/Bcl-2, ATP depletion, and Ca^2+^ overload), DNA damage, cell cycle arrest, and apoptosis. The activation of the p53 pathway, MAPK pathway, JAK/STAT pathway, and AKT pathway is involved in mycotoxin-induced apoptosis. In addition, mycotoxin can directly induce necrocytosis.

The activation and immune responses of astrocytes have been suspected of augmenting proinflammation, calcium imbalance, mitochondrial damage, and the accumulation of oxidative stress products such as reactive oxygen species (ROS), which tend to expand neuronal deterioration and contribute neuronal death [52, 53]. Glial fibrillary acidic protein (GFAP) generated in cell body of astrocytes has been recognized as the activation marker protein of astrocytes (Figure 2) [54]. Previous evidence has suggested that another protein, matrix metalloproteinase 2 (MMP2) with different specific expression pattern [55], played protective role against heavy metals or drug toxicity in astrocytes [56]. During the process of chemical, degenerative, or mechanical injury repair in the brain, the activity, proliferation, and function of astrocytes were influenced by the changes of the expression of GFAP and MTs. Therefore, GFAP and MTs were not only important factors for the stabilization and tight junctions of BBB but also represented the severity of astrocyte activation directly and indirectly, respectively [57]. It has been confirmed that the activation of astrocytes could protect neurons against oxidative stress which occurred in neural stem cells (NSCs) in early progenitor cells of weaned mice infected with T-2 toxin and reduced transient neural disconnection in the hippocampus tissue. Relevant research has observed a significant increase in the number of MT-I/II+ positive astrocytes in various brain regions, and this protective mechanism was suggested as a toxic target of T-2 toxin [58]. Early experiments have shown that astrocyte activation was rapidly initiated in neurodegenerative injury, accompanied by increasing GFAP expression. Similar findings showed that GFAP activity in the cerebellum of rodents was rapidly enhanced by AFB1, promoting the proliferation of astrocytes and the release of inflammatory factors and neurotoxic substances [59]. After long-term oral administration of 0.025 mg/kg AFB1, the GFAP area percentage in hippocampal CA1 area of male rats was significantly increased compared with that of the control group, due to the activation of the nitric oxide (NO) produced by AFB1. As an endothelial relaxation factor, NO initiated the activation of microvascular endothelial cells, with a subsequent activation of astrocytes. Therefore, as the AFB1 administration went on for 90 days, astrocyte toxicity and damage were induced more severely [60]. Previous published studies have suggested that the increased number of astrocytes could also be a repair mechanism during the brain injury and the number of neurons in hippocampal CA1 region, which was rescued by astrocytes. In contrast, both in vivo and in vitro testing results showed that once aflatoxin was freed, the total number of glial cells and neurons in the frontal cortex would decline [61].

### 2.2. Mycotoxin-Mediated Microglia Activation

Microglia, the resident inflammatory sensors in the brain, have been thought to play a key role in the survival and homeostasis of neurons. Activated microglial cells were inclined to show two phenotypes, including the classic M1 phenotypes with the nerve inflammation and the M2 phenotypes focusing on nerve repair [62]. A growing number of evidences have shown that the changed ratio of M1 to M2 due to the excessive activation of microglia could break the balance between inflammation and repair, causing immune disorders, mediate neuroinflammatory injury, and accelerate the neurodegenerative diseases [63, 64]. 24-hour long exposure to 20 ng/mL AFB1 activated the astrocytes as well as human microglial cells in the form of M1 phenotype; subsequent microglia-mediated inducible nitric oxide synthase (iNOS) upregulation promoted the arising of oxidative stress. Gene expression results indicated that the increasing of mRNA expression levels of TLRS, MyD88, and NF*κ*B was evident under the stimulus of AFB1. The secretion of the proinflammatory factors (TNF-*α*, IL6), as well as granulocyte-macrophage colony stimulating factor (GM-CSF), was also promoted, which threatened other resting microglia cells. Nitric oxide has been verified to be one of signals for microglia to switch from M1 to M2 phenotype, but AFB1 only caused a slight increase in NO and had no tendency to activate M2 phenotype of microglia which explained the critical phenotype M1 [32]. Researchers also found that 48 hours of OTA exposure resulted in upregulation of proinflammatory factors such as IL-1*β* and IL-6, and the positive results of macrophage cells ED1/CD68 markers and microglia M1 activation markers were increased, which in turn intensified the release of proinflammatory cytokines. Moreover, anti-inflammatory factors such as IL-4 and IL-10 activated M2 phenotype of microglia. It has been reported that different types of activated glial cells probably interact with each other. For example, MTI and MTII proteins were downregulated by OTA, and it is noteworthy that the inflammatory response of microglia was rescued by the artificially treatment on MTI and MTII proteins in astrocytes, further indicating that the astrocytes might be able to adjust the trends of the activation microglia [65].

## 3. Oxidative Stress in Mycotoxin-Infected Glial Cells

In vivo and in vitro studies have shown that cellular oxidative stress is the direct mechanism of cytotoxicity caused by mycotoxins and their metabolites. ROS are increased by mycotoxins, causing oxidative stress, and then proteins, lipids, and chromosomes are attacked by ROS. Cell membrane is damaged or even lytic end with the increased lipid peroxidation (LPO) and decreased antioxidants [66]. Not only that, brain cells are vulnerable to oxidative stress injury induced by mycotoxins such as AFB1, OTA, T-2 toxins, DON, and FB1 in food and environment because of numerous oxygen consumption, energy expenditure, and peroxide fatty acids in brain tissue [67]. Furthermore, excessive ROS expression and ion imbalance in brain will be further increased with the injury of glial cells, thus damaging the function of the whole CNS [52, 53].

Mycotoxin-mediated oxidative stress in neuroglial cells has been described in related literatures, suggesting that the imbalance between ROS accumulation and elimination is the major neurotoxic mechanism of T-2 toxin among glial cells. The astrocytes of male pups are able to sense the presence of T-2 toxin infection in mice maternal environment and produce oxidative stress response in vivo, which is proved to be regulated by the significantly increased expression of metallothionein in fetal astrocytes [68]. In addition, an increasing number of experiments has been evidenced that oxidative stress is also crucial to the cytotoxicity of microglia, indicating that microglia exposed to microorganisms can not only promote the generation of ROS but also stimulate the expression of NO synthase to promote the production of RNS, which is also considered as a universal indicator of oxidative stress in astrocytes and microglia [69, 70]. It was known from previous studies that ROS and lipid peroxidation were gradually accumulated over time in the cytoplasm of human astrocytes treated with aflatoxin B1. As a consequent of oxidative stress, mitochondrial permeability and DNA molecule are impaired [71]. In addition, aspartate aminotransferase (AST), alanine aminotransferase (ALT), and lactate dehydrogenase (LDH) were significantly released in the brain after oral administration of 20 ng/mL AFB1 for 30 days, and the connectivity of the BBB was completely destructed after 90 days. The findings of neuronal cell apoptosis, nuclear condensation, cellular vacuolization, neuroinflammation, and spongiform necrosis were also systematically recorded in the late stage of oxidative stress [60, 72]. It has been reported that iNOS mRNA of human microglia was upregulated after 3 hours exposure to AFB1, which further confirmed the role of AFB1in promoting oxidation on CNS [73]. However, the observations of free radical assessment in recent literature showed that the increase of the total amount of free radicals and ROS was not obvious after AFB1 treatment, which reflecting that iNOS was not only a prooxidant molecule but also an important indicator in the process of oxidative stress of glial cells [74]. The oxidative stress of normal astrocytes was initiated by 48-hour exposure to OTA, characterized by a reduction of regulatory factors of oxidative stress response [65]. Several researchers treated the serum-free aggregating brain cells with noncytotoxic concentrations of OTA and analyze the results of the quantitative reverse transcription polymerase chain reaction (QRT-PCR). They found that peroxisome proliferator-activated receptor-*γ*, heme oxygenase-1, and iNOS in astrocytes were significantly upregulated, while expressions of glial fibrillary acidic proteins were attenuated [75]. In vitro investigation, oxidative stress was also found to promote FB1-induced neural death pathway because of the role in inducing the cytotoxicity of human U-118MG glioblastoma through the production of ROS, the initiation of lipid peroxidation, and the reduction of glutathione [76]. Despite the evidence that the oxidative stress depends on more than 100 mmol FB1 and 2 to 6 days of exposure and may require some stimulation of indirect mechanism, the oxidative stress damage in glial cells will clearly be evoked by certain dose and duration of exposure. The metabolic changes of sphingomyelin (SL) caused by FB1 can be able to coordinate with oxidative stress to promote cell death and carcinogenesis on this basis [77].

## 4. Mitochondrial Dysfunction Mediated by Mycotoxins

Turning enough adenosine triphosphate (ATP) into chemical energy is essential for cell survive. While mitochondria, the “cellular energy plant” in the brain provides a constant supply of ATP via oxidative phosphorylation (OXPHOS) [78]. Mitochondria can also prevent excessive calcium accumulation in nerve cells and play an important role in the regulation of apoptosis. It has been proved that the regulation of ATP and calcium in glial cells was disrupted as a consequence of mitochondrial dysfunction triggered by mycotoxins, which was also vastly involved in the free radicals, DNA damage, cell apoptosis, and necrosis (Figure 2) [79]. The ability of trichothecenes to inhibit mitochondrial protein translation directly has been exposed. For instance, T-2 toxin-mediated oxidative stress and apoptosis of nerve cells both associated with mitochondrial disorders has been shown in previous studies [80]. Recently, through the 3(4,5-dimethylthiazolyl-2)2,5-diphenyl tetrazolium bromide (MTT) assay signal, scientists found a dose-dependent decrease in mitochondrial activity of mouse microglia (BV-2) after the FB1 (8 mM to 50 mM) infection, and the active numerical value was significantly lower than the control group [81]. It was also found that the ROS produced by the primary mouse astrocytes derived from the impaired mitochondrial function perturbated by FB1. Furthermore, the mitochondrial electron transport chain suppression, mitochondrial membrane decline, and cellular respiratory inhibition eventually induced ROS explosion, measured by real-time imaging and specific inhibitor tests [82]. The oxidative phosphorylation defects of mitochondria have been investigated in AFB1-related tests, and the results showed that DNA damage was parallel to alteration of mitochondrial activity and function. ATP depletion caused by mitochondrial maladjustment was involved in glial cytotoxicity induced by AFB1. For example, in the process of apoptosis in human microglia induced by AFB1 (20 ng/mL) at 24 postexposure hours (PEH), the decreased level of ATP was demonstrated by using firefly luciferase assay [32].

Through the balance between intracellular and extracellular calcium ion (Ca^2+^) signals, astrocytes recycle and release various neurotransmitters such as glutamate and *γ*-aminobutyric acid (GABA) and ensure synaptic stability [83]. It is known that Ca^2+^ depends on ion pumps and Ca^2+^ channels (transient receptor potential channels, L type calcium channel, inositol triphosphate receptor, Ca^2+^-ATP) to enter the neurocyte, and mitochondrial dysfunction-mediated Ca^2+^ migration is accomplished by mitochondrial calcium uniporter [84]. Therefore, the injured astrocyte, depolarized mitochondrion, and the decline of mitochondrial membrane potential (MMP) induced calcium overload, which occurs in the process of AFB1-mediated astrocyte proliferation testing. Interestingly, the proliferation of astrocytes was subsequently rescued after the inhibition of Ca^2+^ levels by chelating agents and inhibitors [85]. An imaging of astrocytes exposured to 3-NPA in vitro has showed an excessive Ca^2+^ overload that caused astrocyte destruction [86]. Furthermore, OTA decreased MMP in human astrocytes, increased the concentration of Ca^2+^ in mitochondria and cytosol, upregulated the proapoptotic factors Bax and P53, and increased the expression level of urokinase plasminogen activator receptor (PLAUR) mRNA [87]. The literature has shown that the calcium influx was in accordance with oxidative stress. For example, transient receptor potential channels and mitochondrial calcium transporters in the astrocytes were activated by excessive ROS, exhibiting the inhibition of the cell survival [88].

## 5. The Inhibition of Glial Cell Viability Caused by Mycotoxins

Mycotoxins have been identified in recent studies as reducing the survival of glial cells in the CNS. The results of neurotoxic correlation assessment of AFB1 suggested that high dose of AFB1 significantly reduced microglial activity in a dose- and time-dependent manner. Treatment with 40 *μ*g/mL AFB1 caused about 50% loss of living glial cells in mice, while AFB1 infection with 1 *μ*M reduced the viability of zebrafish embryos by 66% [85]. The sensitivity of astrocyte viability to mycotoxins has also been demonstrated in previous literatures. For example, murine microglia and astrocytes developed cell necrosis and a decreased number of living cells as a result of in vitro stimulation with FB1 at doses higher than 25 mM [81]. Continuous infection of FB1 in the concentration range of 1-40 mM induced a decrease in astrocyte maturation and survival rate during 25-35 d [89], with significant DNA damage at 48 h, 72 h, and 6 d posttreatment [90]. In addition, about 35% of rat C6 glioma cells died after being treated with FB1 (9 *μ*mol) and incubated for 24 h [91]. In addition, 30% of mice with 3-NPA injection produced abnormal gait, tremor, and somnolence. The swollen and disintegrated striatal astrocytes were observed by electron microscopy [86]. Furthermore, 10 nM T-2 toxin reduced the viability of the astrocyte population by 70% at 24 hours after incubation, and 24 nM T-2 toxin inhibited 50% of cells at 48 hours after exposure, determining the IC_50_ value. It should be emphasized that the comparison of the IC_50_ values of primary astrocytes with those of other primary cells indicated that CNS was susceptible to the toxicity of T-2 toxin [23]. Recently, the sensitivity of different nerve-related cells to mycotoxins has been compared and recognized. The LC50 of NPCs, microglia, and astrocytes in 24-hour AFB1 exposure was 75,000 ng/mL, 50,000 ng/mL, and 40,000 ng/mL, respectively, indicating that the cytotoxic priority of the three types of cells was different [73]. In the test of DON interfered with primary enriched cultures, different glial cells had different response degrees to DON. The IC_50_ of astrocytes was 31 *μ*M, 119 times higher than the value of 0.259 of microglia. Similarly, human astrocyte IC_50_ (50 *μ*M) was more than 12 times that of human microglia IC_50_ (4.1 *μ*M). The microglial cells were also found higher sensitivity in the mixed glia cells coculture system. Immunofluorescence and MTT results showed that DON with a concentration less than 25 *μ*M was resisted by astrocytes. Nevertheless, microglia (ox-42 positive cells) were selectively killed when DON was less than 10 *μ*M [92].

Studies related to cell cycle arrest have also revealed the reasons for the decreased survival rate of glial cells after continuous exposure to mycotoxin. Flow cytometry analysis showed that proliferation of human astrocytes treated with AFB1 was inhibited at the sub G0/G1 stage, which was correlated with the upregulated Bax, Bak, and cytochrome c (Cyt-c), and the proliferation activity of astrocytes was also regulated by the AKT and MAPK signaling pathways [85]. In addition, the sub G0/G1 cell cycle arrest characterized by mRNA downregulation of CCND1, CCNE1, CDK4, and MYC was also observed in OTA infected astrocytes, accompanied by the reduced secretion of neurotransmitters such as norepinephrine, dopamine, and serotonin [87, 93]. Unexpectedly, there was no correlation between the antiproliferation effect of FB1 at a concentration of 2.5 mM on mouse microglia and the cell cycle arrest. Although FB1 has previously been reported to inhibit the proliferation of other cell types through cell cycle arrest, in mouse microglia, it exhibited a unique feature independent of cell cycle arrest [94, 95].

## 6. Apoptosis and Necrosis in Mycotoxin-Treated Glial Cells

So far, apoptosis and necrosis have been explained as the two main indicators of mycotoxin-mediated glial toxicity in vitro (Figure 2). The necrosis and hyperplasia occurred in brain according to immunohistochemical results with the action of AFB1 [96]. It has been reported that AFB1 initiated human astrocytes mitochondrial-dependent apoptosis pathway by inducing a dose-dependent decrease of MMP, an overexpression in proapoptotic proteins such as Bax, Bak, and Cyt-c and a decrease in the expression of phosphorylated Bcl-2 [85]. Microglial apoptosis mediated by low doses of AFB1 (20 ng/mL) was also demonstrated by bioluminescence-based methods. The expression of caspase-3 and caspase-7 increased at 24 PEH, the apoptosis-related genes such as p21 and p53 were significantly elevated, and the phosphatidylserine transferred to the outer surface of the plasma membrane. Simultaneously, apoptotic bodies were formed, and the proportion of apoptosis (annexin V+) increased [97]. Besides, abnormal fluctuations in the expression of Cxcr4 gene were found at different time points in response to AFB1 [98]. It has been reported that the treatment of 1 *μ*M T-2 toxin for 24 h significantly enhanced the activity of caspase-3 in astrocytes, activated the JAK/STAT3 pathway, and eventually triggered apoptosis [68]. In addition, previous detections have revealed that T-2 toxin was time-dependently accumulated in T-2 incubated glial cells. For example, the cumulative concentration of T-2 toxin in astrocytes reached 200 *μ*M after treated with 10 *μ*M T2 toxin for 15 minutes. Subsequently, the absorption of T-2 toxin by human astrocytes reached a peak at 320 *μ*M after 1-hour long incubation, which was about 30 times of the concentration of T-2 toxin in the peripheral medium. Likewise, it was also found that HT-2, the main metabolite of T-2 toxin, also generated in human astrocytes and reached the maximum concentration of 150 *μ*M after 6 hours of exposure, indicating that the apoptotic pathway of primary astrocytes was produced by cooperation of T-2 and HT-2 toxins [23]. Moreover, researchers have determined the apoptosis in 3-NPA treated primary astrocytes and BV2 cells, and simultaneously, these cells expressed huntingtin. In turn, 0.5 mg/kg *α*-melanocyte stimulating hormone (*α*-MSH) could protect cells via promoting brain derived-neurotrophic factor (BDNF) and peroxisome proliferator-activated receptor-*γ* (PPAR-*γ*) expression [99]. In addition, researchers found that OTA triggered astrocytotoxicity through mitochondria-dependent apoptosis which was related to the increased phosphorylation of proteins in AKT and MAPK signal transduction pathway (e.g., JNK and ERK) [100]. In the other side, the role of mycotoxins in inducing direct or secondary glial cell necrosis, which was another way of nonneuronal cell death, has been speculated. For example, the death of nonapoptotic necrotizing cells occurred 8 days after 50 *μ*M FB1 infection in BV-2 and murine precursor astrocytes, as confirmed by lactate dehydrogenase release assay and annexin V/PI staining observations [81]. Based on the evidence of necrosis caused by FB1 and the past data on FB1 infected hamster ovarian cell test, researchers mechanistically speculated that the proapoptotic signals of such cells were possibly not vulnerable to boost by FB1. Instead, the necrotic death mode was involved in cell loss triggered by FB1 [101].

## 7. Inflammation/Immune Deregulatory in Glial Cells

**Figure 3:**
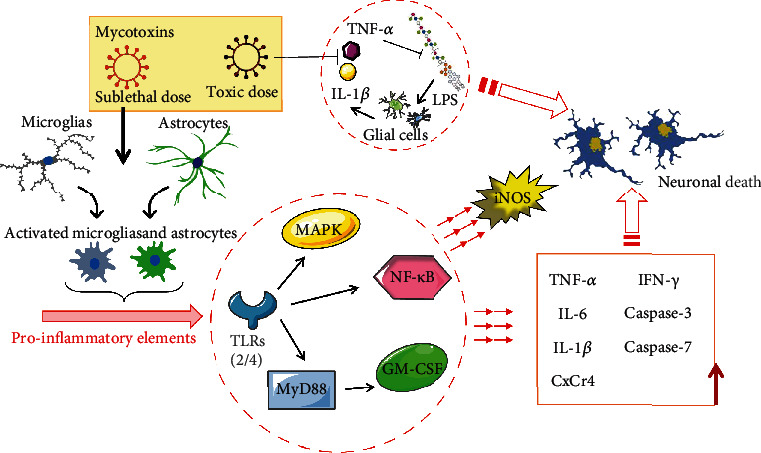
Mycotoxin-induced inflammation/immune deregulatory in glial cells. Sublethal dose of mycotoxins can activate microglia and astrocytes and promote proinflammatory element release. Mycotoxin activates MAPK, NF-*κ*B, and MYD88/GMCSF pathways through toll-like receptors (TLRs) to promote the release of iNOS and inflammatory cytokines, including TNF-*α*, IL-6, IL-1*β*, CxCr4, IFN-*γ*, caspase-3, and caspase-7, and ultimately leads to death of neuronal cells. On the other hand, toxic dose of mycotoxin can reduce microglia's sensing for LPS through cytotoxic effects, leading to the risk of decreased anti-infection efficiency with the downregulation of TNF-*α* and IL-1*β*.

Activated microglia cells and astrocytes both normally have the ability to release soluble factors for neuroinflammation, and their immune response has been considered to be essential for neuroinflammation [102, 103]. In general, inflammation could be produced and developed with injury, infection, and toxin stimulation. Although transient neuroinflammation may contribute to the elimination of pathogenic microorganisms, repair of brain infections, and protection of the nerve tissue, the chronic neuroinflammatory signal transduction could also potentially translate to the development of neurodegenerative disease, suggesting that inflammatory activation of glial cells could have biphasic effects [13, 104]. For example, astrocytes tend to maintain the homeostasis of brain function under normal physiological conditions, but they could be abnormally activated with injury or pathological interference, releasing various inflammatory factors and promoting the course of neurological diseases [105]. In addition, the immune effector cells of the CNS, microglia, normally played an important role in immune regulation, including sensing harmful stimuli in the environment, swallowing debris and abnormal proteins, digesting apoptotic neurons, and performing antigen-presenting function. On the other hand, microglia have also been recognized as an internal cause of pathologic neuroinflammation, exacerbating a variety of neurodegenerative diseases, including Parkinson's disease, Alzheimer's disease, amyotrophic lateral sclerosis, and multiple sclerosis [106–108]. In the latest literature, activation and proinflammatory response/immune dysregulation of murine microglia exposed to low quantity of AFB1 have been evaluated. Functional toll-like receptors (TLRs), as a key immunosensor of the CNS, have been known as one of the characteristics of the immune activity of glial cells, as well as the proinflammatory events in the brain in the stimulation of the external environment. TLR2 was overexpressed in 20 ng/mL AFB1-exposed microglia after 1 hour and triggered a prophase cascade, including MyD88, NF*κ*B2, TNF-*α*, IL-6, chemokine receptor (CxCr4), and caspase-3/7, which were upregulated at gene and protein levels. The level of TLR4 then increased after 5 hours, further triggering the secretion of iNOS and NF-*κ*B2 [73]. GM-CSF could aggravate inflammatory cell infiltration in mammalian brain, glial cell proliferation, and central nervous system immune disorder. A vitro evaluation indicated that MyD88 and TLRs stimulated the secretion of IFN-*γ* and GM-CSF in activated microglia following exposure to AFB1, thus recruiting other resting microglia [32]. In AFB1-initiated proinflammatory microenvironment, astrocytes also increased the loads of TNF-*α*, IL-1, and IL-6 through TLR expression (IL-6 was the most upregulated) to exacerbate inflammation and injury, which was confirmed by cytokine expression profiles [73]. In addition to AFB1, repetitive OTA-derived stimulation has been believed to enhance neuroinflammation as visualized by an increase of activating glial cells and proinflammatory signals, such as iNOS, IL-1*β*, TNF-*α*, and IL-6, which were increased after 24 hours of exposure. M1 microglial cells were fully activated on day 10 of exposure, and ED1/CD68 positive cells and isolectin B4-labeled cells were significantly increased. Under the same conditions, GFAP, MTI, and MTII in astrocytes, that were known to be normally elevated in injury, were downregulated in the AFB1 environment, and the expression of vimentin in astrocytes was atypically increased [65]. Furthermore, the mechanism of 3-NPA-induced TNF-*α*, IL-1*β*, and IL-6 in mRNA levels was related to Nrf2 expression, which was the therapeutic target in the treatment of Huntington's disease [27]. OTA was also suspected to influence the expression of brain inflammation-related genes through astrocytic cytoskeletal changes in another study, and MTI/MTII was also suggested to be involved in the regulation of OTA-induced neuroinflammation [109]. It has been well documented that microglia could be activated by bacterial lipopolysaccharides (LPS), then release proinflammatory factors and produce ROS and reactive nitrogen (RNS), causing damage and loss of peripheral neurons and glial cells. Therefore, the inhibition or promotion of above process could be sense for the study of degenerative neurological diseases and pathologic neuroinflammation [110]. Remarkably, DON possibly has a double side on the secretion of TNF-*α* and iNOS in microglia treated with LPS. For example, DON (sublethal dose) of less than 100 nM promoted the inflammatory effects through MAPK and NF-*κ*B pathways, thereby increasing neuronal damage caused by infection, while DON (toxic dose) of IC_50_ reduced microglial sensing for LPS through cytotoxic effects, leading to the risk of decreased anti-infection efficiency [92, 111]. Similarly, PCR results demonstrated that the expression of proinflammatory molecules including TNF-*α* and IL-1*β* was downregulated in the mixed culture of glial cells incubated with FB1 for 6 h or 24 h [81, 112]. Judging from the available evidence, mycotoxins have been suggested to enhance or inhibit the production of proinflammatory factors in glial cells at the mRNA and protein levels, which in turn provide the possibility of brain instabilities, neuroimmune disturbances, and neurodegenerative diseases (Figure 3).

## 8. Failure of Glutamate Clearance in Glial Cells

**Figure 4:**
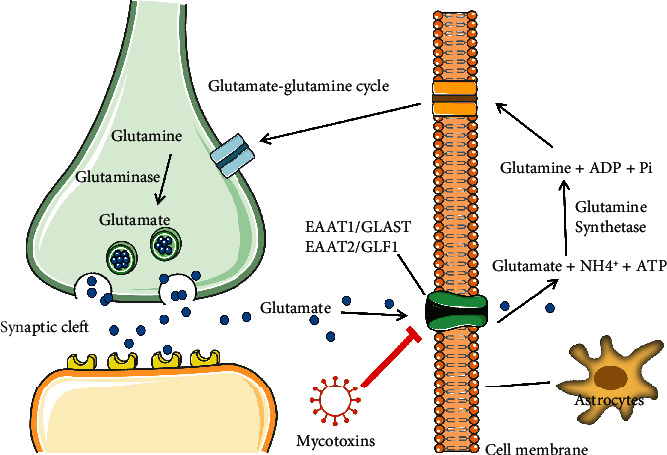
Mycotoxins inhibit glutamate clearance in glial cells. Mycotoxin-mediated astrocyte injury inhibits glutamate uptake and leads to a large accumulation of extracellular glutamate, which eventually induced excitotoxicity, functional impairment, or death of nerve cells.

It has been known that the survival of neurons depends on astrocytes, partly because the latter responds to an overdose of neurotransmitters through their receptors and reabsorb some of the neurotransmitters to end the synaptic process in time [113]. The “glutamate-glutamine cycle” has been known as the main metabolic coupling pathway between astrocytes and neurons. The glutamate has been considered both as the primary excitatory neurotransmitter in the CNS of mammals and a potential neurotoxin whose excitatory toxicity may lead to the death of nerve cells. To remain the sensitivity of the synaptic transmission, astrocytes timely ingest and buffer glutamate through high-sodium-dependent excitatory amino acid transporters (EAAT) including EAAT1/GLAST and EAAT2/GLT-1. Glutamate is then converted into glutamine by the enzyme glutamine synthetase (GS) in astrocytes and released extracellular, where it will be reabsorbed by neurons into a new cycle [114]. In addition to terminating synaptic transmission, glutamate clearance by astrocytes could also prevent excitotoxicity, functional impairment, or neuron death due to high level of glutamate [115, 116]. In the early stage, researchers selectively inhibited the expression of glutamate vector in astrocytes by using antisense nucleic acid technology, leading to astroglial reactivity characterized by dramatically increased concentration of extracellular glutamate, and the neurons eventually deteriorated due to excitotoxicity. In addition, it has been verified that neurons release large amounts of glutamate during cerebral anemia, and astrocytes subsequently absorb glutamate to protect neurons from damage. In infection, inflammation, hypoxia, ischemia, trauma, and various pathologies like Alzheimer, the ability of GS to recover and convert glutamate was suppressed, leading to neuroexcitatory toxicity and neuronal death [116, 117]. Similarly, mycotoxin-mediated astrocyte injury also inhibited the uptake of glutamate and induced a large accumulation of extracellular glutamate. For example, DON decreased the expression of EAAT on the plasma membrane of astrocytes in time-dependent manner at 1 and 10 *μ*M, thereby inhibiting the function of taking in L-glutamate from the synaptic gap. The IC_50_ of this inhibition was 50 nM, which had no correlation with the DON toxic-mediated decline in astrocyte activity [92]. In recent reports, OTA affected the membrane insertion state of EAATs in cell surface of primary astrocytes, and the expression of glutamate transporter 1 (GLT1) was also downregulated by OTA infection, resulting in the failure of L-glutamate capture in the nervous system [118]. Now we know that these mycotoxins could inhibit manipulation of neurotransmitters in limited findings, suggesting that all neural activity associated with information transmission could be regulated by mycotoxins in our environment through the glial cells (Figure 4).

## 9. Conclusions

On the basis of existing studies in regard to mammalian nervous system diseases, the important position of glial cells in the CNS has been increasingly recognized. The homeostasis of the brain environment, the transmission of synaptic information, the integrity of the BBB, and the survival of neurons are all maintained and monitored by glial cells, including microglia and astrocytes. Once the viability and function of glial cells were lost, neurological diseases and neuronal death characterized by abnormal glial cells would be triggered. It is tempting to liken the glial cells, led by astrocytes and microglia, to the umbrella of the CNS, where exogenous stimuli with neurotoxic effects could more or less destroy these guardian, causing neurological damage. In view of the more frequent occurrence of mycotoxin overdose and poisoning in the consumption of substandard food and feed, mycotoxins have been gradually included in the research of potential neurotoxins. Recent studies have found that mycotoxins such as AFB1, DON, T-2, OTA, FB1, and 3-NPA could cross the BBB, accumulate in the brain, and disrupt the normal order of CNS. In addition, glial cells, especially astrocytes and microglia, have been found to be significantly affected by the toxicity of mycotoxins, which possibly because they are themselves immunosensors and inflammatory effector cells. Compared with other neural accessory cells, these two glial cells tend to be more sensitive to mycotoxins and exhibit toxic effects more obviously.

**Figure 5:**
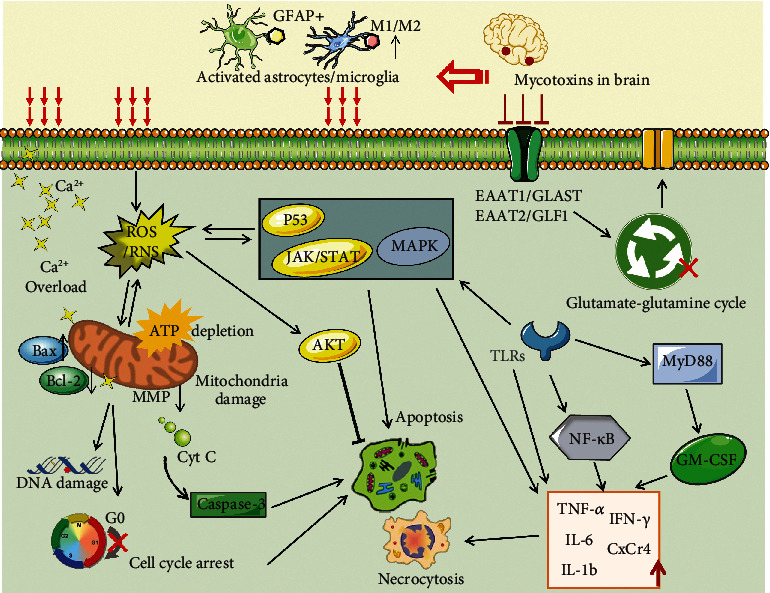
Molecular mechanisms of mycotoxin-induced neurotoxicity in neuroglia cells: (i) ROS and RNS were accumulated in oxidative stress damage; (ii) mitochondrial function was impaired, aggravating ATP depletion and calcium imbalance; (iii) the ability to buffer transmitters such as glutamate was inhibited, inducing neural defects; (iv) the viability of glial cells was decreased, cell proliferation was inhibited at the G0/G1 stage, and the release of proinflammatory factors changed dramatically, resulting in apoptosis or necrosis; (v) the activation of the p53 pathway, MAPK pathway, JAK/STAT pathway, and AKT pathway is involved in mycotoxin-induced apoptosis.

As illustrated in Figure 5, astrocytes and microglial cells were activated by mycotoxins in different forms; the expression of GFAP and MMP2 in the former was adjusted, and the ratio of M1 and M2 activation phenotypes in the latter was changed. Whatever, excessive activation of glial cells could bring out subsequent glial cell toxicity effect: (i) ROS and RNS were accumulated in oxidative stress damage; (ii) mitochondrial function was impaired, aggravating ATP depletion and calcium imbalance; (iii) the ability to buffer transmitters such as glutamate was inhibited, inducing neural defects; (iv) the viability of glial cells was decreased, cell proliferation was inhibited at the G0/G1 stage, and the release of proinflammatory factors changed dramatically, resulting in apoptosis or necrosis of the cells eventually; (v) the activation of the p53 pathway, MAPK pathway, JAK/STAT pathway, and AKT pathway is involved in mycotoxin-induced apoptosis. According to the related research, we can analyze that even low-dose food-derived mycotoxins could mediate brain damage and neuronal death through glial cytotoxicity and promote the occurrence of pathological neuroinflammation and neurodegenerative diseases. It is important to note that the incidence of neurodegenerative diseases is increasing, which affects the normal neurogenic behaviour of human beings. Neurodegenerative diseases are closely related to environmental changes, and in fact, mycotoxin contamination has been one of the major problems caused by environmental changes. Therefore, mycotoxin-induced nerve damage is expected to become the key to deal with to the trend of neurodegenerative diseases. Of course, these evidences could further guide researchers to explore the connections between glial cells and other important components in the common microenvironment of CNS, such as the complex regulatory mechanisms between glial cells and various neurons that are simultaneously exposed to mycotoxin. In addition, the combined neurotoxicity of different mycotoxins in vivo and in vivo may also be the next research target, given that their synergistic mutagenesis has been demonstrated in other cells. Most importantly, the glial toxicity of mycotoxins reminds us to develop neuroprotective agents targeting glial cells in order to effectively respond to neurological diseases, such as Alzheimer's, Parkinson's, and Huntington's diseases. In conclusion, understanding the link between mycotoxins and glial injury could help us more comprehensively and effectively assess the risks of animal feed and food and also provide data support for drug new targets in CNS, so as to overcome more clinical troubles. Given the difficulty in completely avoiding mycotoxins in food crops and the absence of visible warning labels on raw materials and consumer goods, real-time and efficient supervision on food security remains the primary tasks of ensuring human and animal health.
